# The Antioxidant Protein Peroxiredoxin 4 Is Epigenetically Down Regulated in Acute Promyelocytic Leukemia

**DOI:** 10.1371/journal.pone.0016340

**Published:** 2011-01-20

**Authors:** Karishma K. Palande, Renee Beekman, Lotte E. van der Meeren, H. Berna Beverloo, Peter J. M. Valk, Ivo P. Touw

**Affiliations:** 1 Department of Hematology, Erasmus University Medical Center, Rotterdam, The Netherlands; 2 Department of Clinical Genetics, Erasmus University Medical Center, Rotterdam, The Netherlands; Emory University, United States of America

## Abstract

The antioxidant peroxiredoxin (PRDX) protein family comprises 6 members, which are implicated in a variety of cellular responses, including growth factor signal transduction. PRDX4 resides in the endoplasmic reticulum (ER), where it locally controls oxidative stress by reducing H_2_O_2_ levels. We recently provided evidence for a regulatory function of PRDX4 in signal transduction from a myeloid growth factor receptor, the granulocyte colony-stimulating factor receptor (G-CSFR). Upon activation, the ligand-induced G-CSFR undergoes endocytosis and routes via the early endosomes where it physically interacts with ER-resident PRDX4. PRDX4 negatively regulates G-CSFR mediated signaling. Here, we investigated whether *PRDX4* is affected in acute myeloid leukemia (AML); genomic alterations and expression levels of *PRDX4* were investigated. We show that genomic abnormalities involving *PRDX4* are rare in AML. However, we find a strong reduction in *PRDX4* expression levels in acute promyelocytic leukemia (APL) compared to normal promyelocytes and different molecular subtypes of AML. Subsequently, the possible role of DNA methylation and histone modifications in silencing of *PRDX4* in APLs was investigated. We show that the reduced expression is not due to methylation of the CpG island in the promoter region of *PRDX4* but correlates with increased trimethylation of histone 3 lysine residue 27 (H3K27me3) and lysine residue 4 (H3K4me3) at the transcriptional start site (TSS) of *PRDX4*, indicative of a bivalent histone code involved in transcriptional silencing. These findings suggest that the control of G-CSF responses by the antioxidant protein PRDX4 may be perturbed in APL.

## Introduction

G-CSF induces the proliferation, survival and neutrophilic differentiation of myeloid progenitor cells [1]. This response depends on the balanced activation and subsequent attenuation of signaling pathways linked to the G-CSFR, which is to a major extent controlled by lysosomal routing of the activated G-CSFR complex [2], [3]. Signal attenuation of the G-CSFR is compromised by mutations causing truncations in the cytoplasmic domain of the receptor, found in severe congenital neutropenia (SCN) patients showing disease progression to acute myeloid leukemia (AML) [4], [5], [6]. These truncated G-CSFR forms are defective in internalization and lysosomal routing, which plays a major role in the abnormal signaling function of these receptor mutants.

Another important feature of the G-CSFR truncation mutants is that their activation results in elevated levels of reactive oxygen species (ROS) relative to the wild type G-CSFR [7]. ROS have been shown to modulate growth factor signal transduction, due to inactivation of oxidation sensitive protein and lipid phosphatases [8], [9]. Increased concentrations of intracellular ROS, however, have been implicated in DNA damage as well as in damage to proteins and lipids. Hence it is of importance to maintain steady state levels of ROS. PRDX4 is an antioxidant protein which resides in the endoplasmic reticulum. It is not only responsible for maintaining steady state levels of ROS but also for reactivation of oxidized phosphatases [10], [11]. Our recent findings suggest that PRDX4 interacts with a C-terminal region of the G-CSFR, a region lacking in the truncated G-CSFR found in SCN/AML. PRDX4 was shown to attenuate G-CSFR signaling dependent on the integrity of its redox active thiol group (Palande et al, manuscript submitted). Supporting a possible involvement of *PRDX4* in leukemogenesis, a chromosomal translocation, t(X;21)(p22;q22) has been reported in a case of AML resulting in a *PRDX4-RUNX1* fusion transcript [12].

We have investigated whether the *PRDX4* locus is associated with translocations and mutations in AML and whether its expression levels are altered. We report that chromosomal translocations involving the *PRDX4* locus are rare in AML. In addition, we did not detect single nucleotide variations in the *PRDX4* coding region in a large panel of AML and MDS patients. On the other hand, we found that *PRDX4* expression is significantly decreased in APL, a subset of leukemias that is characterized by hyper responsiveness to G-CSF [13]. Reduced *PRDX4* expression in APL is associated with a bivalent histone methylation mark, i.e., the combination of repressive histone methylation mark H3K27me3 and the activating histone methylation mark H3K4me3, at the TSS of *PRDX4* but not with DNA methylation of CpG islands in its promoter region.

## Results

### Genetic abnormalities affecting the *PRDX4* coding region are rare in MDS and AML

Previously, a case of AML with a t(X;21)(p22;q22) has been reported in which the *PRDX4* gene located on Xp22 was fused to *RUNX1* at 21q22, resulting in a *RUNX1-PRDX4* fusion transcript [12]. In a cohort of AML and MDS patients karyotypically analyzed in our institution between 1990 and 2008, we found 9 cases with chromosomal abnormalities involving Xp21/p22 by standard banding techniques. These cases were further studied for possible rearrangements in the *PRDX4* locus using FISH with a *PRDX4* break apart probeset, but no translocations, deletions or other gross rearrangements were detected. Subsequently, we screened cDNA from 65 MDS patients and 113 AML patients for possible mutations or polymorphisms, but no mutations in the *PRDX4* coding region were detected in these samples. Thus, our data confirms that genomic aberrations affecting the *PRDX4* coding region are rare in MDS/AML and so far confined to the one reported case [12].

### PRDX4 is down regulated in APL

**Figure 1:**
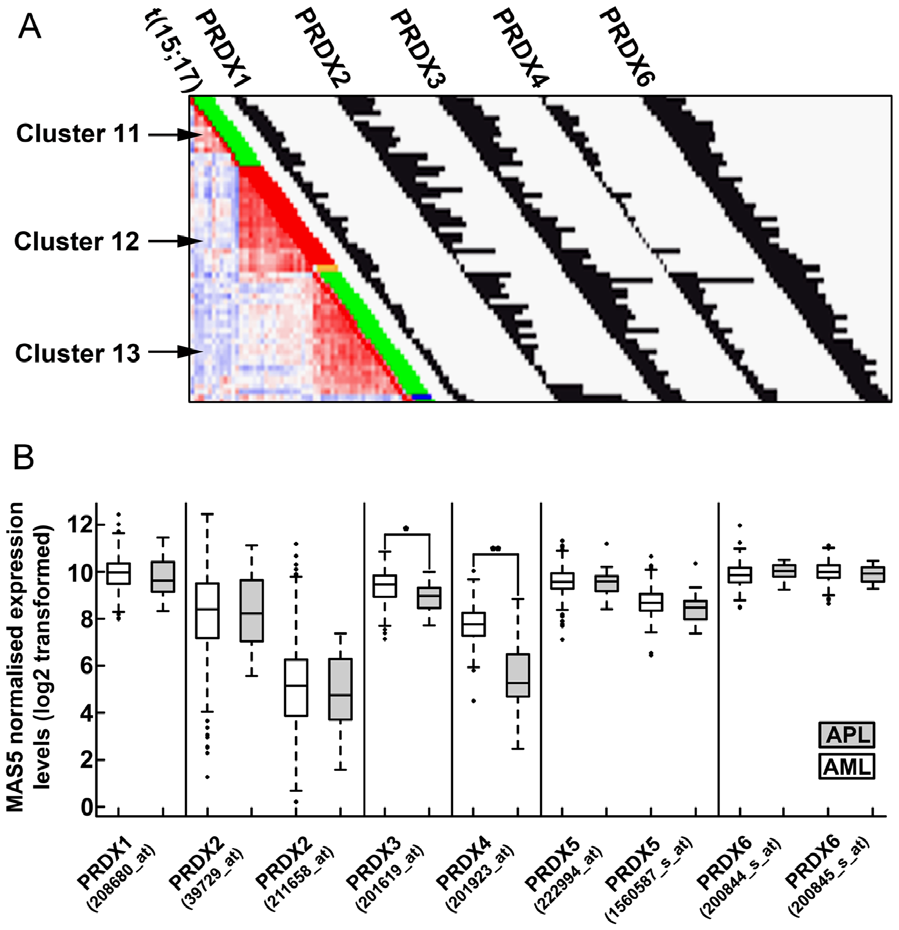
Expression of PRDX transcripts in AML and APL. **A**. Graphical representation of expression of PRDX family members in APL patients clustered based on expression of ∼2000 genes as described [24]. Cluster 12 is exclusively formed by APL patients, as indicated by the red bars indicating the presence of t(15;17). Cluster 11 comprises AML patients with normal karyotype and an underlying NPM1 mutation. Cluster 13 is formed by AML patients with t(8;21). Histograms represent MAS5-normalized expression values. **B**. Expression levels of the peroxiredoxin gene family in APL (n = 22) were compared to transcript levels in AML (n = 439). Significant differences were calculated using a Wilcoxon test. *  =  p-value <0.001, **  =  p-value <0.0001.

**Figure 2:**
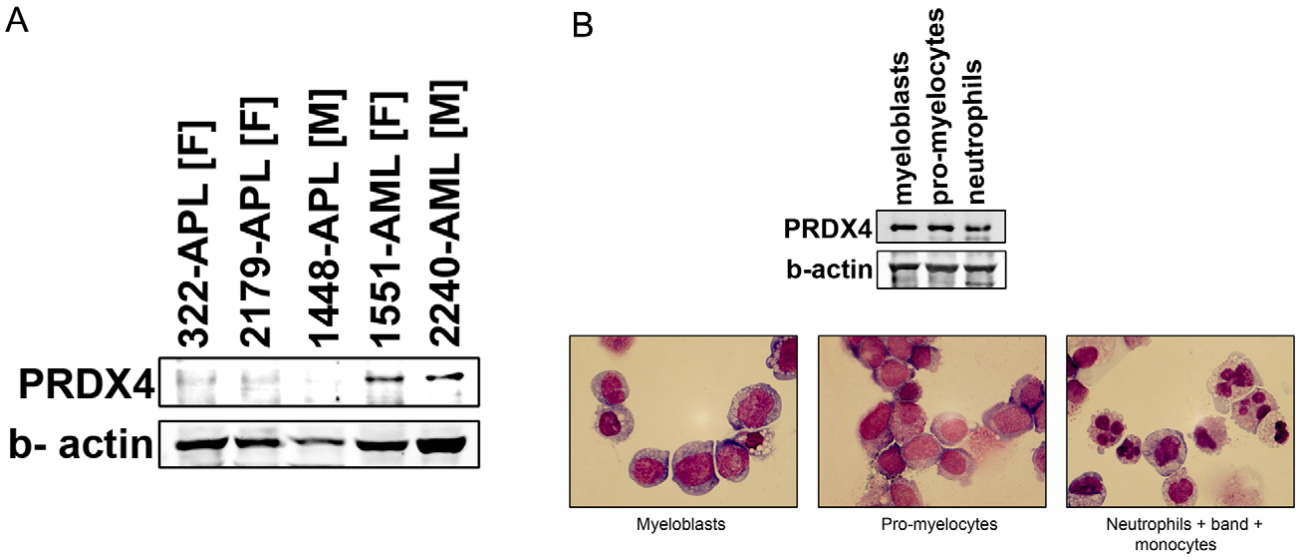
Reduced PRDX4 protein and transcript levels in APL. **A**. Lysates of APL and AML samples were analyzed for PRDX4 protein levels. Because human *PRDX4* gene is located on the X-chromosome, male samples and female samples were compared with male and female AML control samples respectively to rule out the possibility of X-inactivation involvement. Patient number followed by F indicates female sample while patient number followed by M indicates male sample. **B. Upper panel**: Western blot analysis of PRDX4 expression in different normal bone marrow fractions (myeloblasts, promyelocytes, neutrophils). **Lower panel**: Micrographs of May Grunwald Giemsa stained fractions of cells used for Western blotting.

Next, we analyzed *PRDX4* expression levels in 461 myeloid leukemia patients measured on gene expression arrays [14]. In the majority of APL patients, PRDX4 levels are below detection levels; on average, *PRDX4* transcript levels were 4 to 5 times lower in APL, relative to other AML samples (Figure 1A and 1B). In contrast, expression of the other *PRDX* family members were not (*PRDX1*, *2*, *5* and *6*) or only marginally (*PRDX3*) reduced in the APL patient cluster (Figure 1A and B). A recent study showed that TNFα-related Apoptosis Inducing Ligand (TRAIL) suppresses the expression of *PRDX4* [15]. We therefore first investigated whether low levels of *PRDX4* expression in APL are associated with high expression of *TRAIL*, but we did not find evidence for such an inverse correlation (Pearson correlation coefficient 0.26–0.35 for 3 different *TRAIL* probe set comparisons, data not shown). Western blot analysis showed that the PRDX4 protein, while readily detectable in AML blast cells with high transcript levels, is low/undetectable in APL cells correlating with the gene expression profiling data (Figure 2A). In contrast, PRDX4 protein levels were not reduced in normal bone marrow-derived myeloblasts, promyelocytes and myelocytes (Figure 2B, upper panel), suggesting that the down regulation of PRDX4 is specific for leukemic promyelocytes. The purity of the sorted fractions of cells used for Western blotting as mentioned above was assessed using cytospins of the individual fractions stained with May GrÜnwald Giemsa (Figure 2B, lower panel).

### Reduced *PRDX4* expression in APL is not due to DNA methylation of CpG islands in the *PRDX4* promoter

PML-RARα recruits DNA methyltransferases such as DNMT1 and DNMT3a and is thereby potentially able to methylate CpG islands in target genes, leading to transcriptional silencing [16]. We asked whether *PRDX4* expression in APLs might be silenced by DNA methylation. Because *PRDX4* is located on the X chromosome, one allele is methylated in female cells. CpG methylation on both alleles in female samples or on one allele in the male APL samples was not observed (data not shown). Thus, no evidence was obtained indicating that DNA methylation of CpG islands within the promoter region contributes to silencing of *PRDX4* in APL cells.

### APL cells have increased levels of H3K27me3 and H3K4me3 at the TSS of *PRDX4*

**Figure 3:**
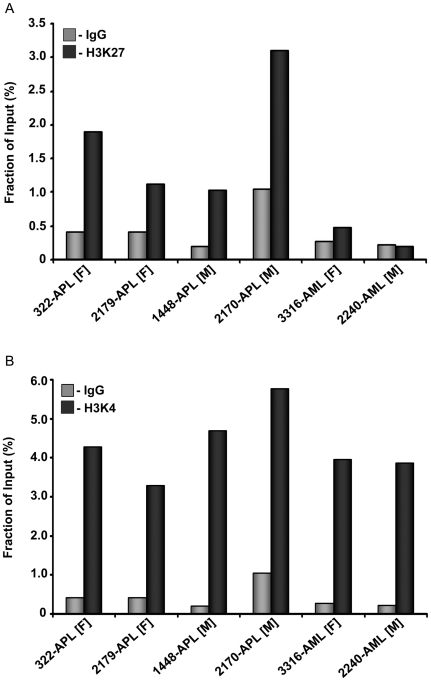
Bivalent H3K27me3 and H3K4me3 marks are present at the TSS of *PRDX4.* ChIP using αH3K27me3 and an IgG control (**A**) and αH3K4me3 and an IgG control (**B**) showing an enrichment of both H3K27me3 and H3K4me3 at the TSS of *PRDX4* in APL cells. To rule out a possible involvement of X-inactivation, and thereby H3K27me3, in silencing of *PRDX4* expression, male APL samples are compared to a male AML control and female APL samples are compared to a female AML control.

PML-RARα recruits the polycomb repressor complex 2 (PRC2) that contains EZH2 as a major effector protein [17]. EZH2 represses gene expression through H3K27me3, leading to an inactive chromatin configuration [18]. ChIPs performed on primary APL cells showed an increased occupation of H3K27me3 at the TSS of *PRDX4* (Figure 3A), which was not observed in AML samples expressing PRDX4. Unlike the H3K27me3 ChIPs, results from H3K4me3 ChIPs showed occupation of the TSS of *PRDX4* by the H3K4me3 methylation mark in both, APLs as well as AMLs (Figure 3B).

## Discussion

The major finding reported here is that expression of the antioxidant protein PRDX4 is repressed in t(15;17) APL harboring the PML-RARα fusion protein. Our data indicate that transcriptional regulation of *PRDX4* is perturbed in APLs because normal promyelocytes do not show reduced PRDX4 protein levels. This reduced expression is linked to a bivalent histone mark, i.e., increased H3K27me3 and H3K4me3, at the TSS of *PRDX4*. Bivalent histone marks provide a switch mechanism shown to be involved in transcriptional regulation of developmental genes; loss of the H3K27me3 mark leads to transcriptional activation whereas loss of H3K4me3 results in permanent silencing [19]. Our data suggest that reduced PRDX4 expression in APLs is caused by maintenance of H3K27me3, which perturbs the bivalent switch. We hypothesized that PML-RARα might be involved in repressing *PRDX4* expression, especially because PML-RARα recruits the polycomb repressor complex (PRC2) that contains the histone methyltransferase EZH2 responsible for H3K27me3 [17]. Wild type retinoic acid receptors (RARs) are known to bind to specific DNA sequences called RA responsive elements (RAREs) and are able to repress transcription by recruiting co-repressor complexes such as SMRT/NCoR/HDAC [20]. The promoter region of *PRDX4* however does not contain any retinoic acid responsive element (RARE) that may be bound by RARα/RXR directly. Another mechanism shown to be involved in suppression of genes via PML-RARα is the modulation of the activity of Sp1 target genes via binding to the transcription factor Sp1 [21]. Six Sp1 sites are present within the region of −1 kb to +1 kb of the TSS of *PRDX4,* to which the PML-RARα fusion protein may bind in a RARE independent manner [21].

To support the hypothesis of PML-RARα-mediated recruitment of PRC2/EZH2 to the *PRDX4* TSS, leading to H3K27me3 repression of the gene, we have attempted to restore PRDX4 expression in primary APL. To achieve this, APL cells were incubated with (combinations of) pharmacological compounds that included the “de-repressing” agents ATRA, the histone deacetylase inhibitor valproic acid and EZH2 inhibitor DZNep, which reduces EZH2 protein levels and thereby the level of H3K27me3 at transcriptional start sites of certain genes [22]. However, down regulation of EZH2 by DZNep, as previously shown by Jiang et al in colorectal cancer cells, was not seen in the APL samples used in our studies. Further studies are required to support a direct involvement of PML-RARα in *PRDX4* down regulation.

Irrespective of the molecular mechanism of transcriptional silencing, a lack of PRDX4 may have a major impact on cellular responses of myeloid cells and contribute to leukemogenesis. PRDX4 resides mainly in the endoplasmic reticulum (ER), where it is thought to neutralize the oxidizing effects of locally produced ROS [23]. This would keep for instance oxidation sensitive ER-resident phosphatases, such as protein tyrosine phosphatase 1b (PTP1B), in an active state. PTP1B has been shown to play a role in down modulation of G-CSFR mediated signaling by dephosphorylating JAK2, STAT3 and the tyrosines of the G-CSFR (Palande et al, manuscript submitted). Predictably, loss of PRDX4 could lead to a reduction of phosphatase activity, providing an explanation for the increased responsiveness of APL clonogenic precursors to G-CSF.

## Materials and Methods

### Human cell samples

All human cell samples were obtained after written informed consent and stored anonymously in a biobank. The study was performed under the permission of the Institutional Review Board of the Erasmus MC, registration number MEC-2008-387. Preparation of leukemia cell samples has been described previously [24]. Normal myeloblasts, promyelocytes and neutrophils were isolated from normal bone marrow samples using fluorescence activated cell sorting (FACS). Erythrocytes were removed prior to FACS sorting, by hypotonic lysis (15 min.), followed by a wash with phosphate buffer saline (PBS). The cells were then resuspended in PBS and incubated with fluorescent dye conjugated antibodies CD10-APC, CD11b-APC-Cy7, CD34-Pe-Cy7 and CD117-PE (Becton Dickinson, NJ) dead cells were excluded using a combination of forward and side scatter and DAPI staining. Cells were sorted with a FACSAria (Becton Dickinson, Becton Dickinson and Company, NJ) on the following criteria: CD34− and CD117+ for promyelocytes; CD34+ and CD117+ for myeloblasts; CD10+ and CD11b+ for neutrophils.

### Mutation analysis of the *PRDX4* coding region

A WAVE device (Transgenomics, Omaha, NE, USA) was used to screen for mutations in the *PRDX4* coding region. cDNA from 113 AML and 65 MDS patients was analyzed. The coding region of *PRDX4* was PCR amplified in 4 separate parts, the length of each part being approximately 300 base pairs with an overlap of at least 60 base pairs between two consecutive fragments. The first part of the PCR was amplified using primers *PRDX4* F1 (5′-CCAAGGGACGTGTTTCTGCG-3′) and primer *PRDX4* R1 WAVE (5′-CTGGCTTGGAAATCTTCGC-3′). The second part was amplified using primers *PRDX4* F2 WAVE (5′-GAGGAGTGCCACTTCTACGCG-3′) and *PRDX4* R2 WAVE (5′-CTGTGAATCAACAGAGCATG-3′). The third part of *PRDX4* was amplified using primers *PRDX4* F3 WAVE (5′-GGTTTTCTTCTTCTACCCACT-3′) and *PRDX4* R3 WAVE (5′-CATTCAGAGTAATTTGTCTTAG-3′). The last part of *PRDX4* was amplified using primers *PRDX4* F4 WAVE (5′-GGACTATGGTGTATACCTAG-3′) and *PRDX4* R1 (5′-CCGTGAACTTTATTGAGAACTTTC-3′).

### FISH analysis

A locus-specific break apart FISH probeset was designed using the BAC clones CTD-2114P24 (chrX: 23457583- 23659210) and CTD-2594J24 (chrX:23638596-23807502). Clone isolation and labeling were performed using biotin-16-dUTP and digoxigenin-11-dUTP (Roche Diagnostics Belgium, Vilvoorde, Belgium) according to the manufacturer's protocol. The FISH analysis was performed as previously described on metaphase preparations. For this, patient samples were cultured and harvested according to standard cytogenetic protocols. Hybridized preparations were digitally imaged and analyzed using Isis (MetaSystems, Altlussheim, Germany). A minimum of 100 interphase nuclei and 5 metaphases were analyzed [25].

### Gene expression analysis

To determine expression of different peroxiredoxin family members (*PRDX1-6*), data of 439 de novo AML and 22 APL samples were used [14]. For each gene, probe sets were determined and the average expression levels in AML and APL were calculated. Significant differences within these two groups were determined with a Wilcoxon test. Multiple testing correction was performed using the Benjamini-Hochberg algorithm. Probesets with an FDR below 0.05 were considered to be differentially expressed in APL compared to AML. To examine a possible inverse correlation between expression of *PRDX4* and *TRAIL*, Pearson correlation coefficients were calculated separately in the APL cases.

### Western blotting and antibodies

Cells were lysed in lysis buffer (20 mM Tris HCl pH 8.0, 137 mM NaCl, 10 mM EDTA, 100 mM NaF, 1% NP40, 10% glycerol, 2 mM Na3VO4 and 1 mM Pefablock SC). SDS-polyacrylamide gel electrophoresis was performed using precast 4–12% bis-Tris gradient gels (Invitrogen, Breda, the Netherlands). Prdx4 mouse monoclonal antibody was purchased from Abcam, (Cambridge, UK), β-actin antibody from Santa Cruz Biotechnology Inc. (Santa Cruz, CA).

### Bisulfite sequencing

Bisulfite treatment of genomic DNA from APL or AML samples was performed using the EpiTect bisulphite kit (Qiagen, Hilden, Germany). PCR was performed on bisulfite treated DNA using methylation insensitive primers *PRDX4* bisulfite seq Fw1 (5′-TTGTTTTTATAGAGTTGGGTAA-3′) and *PRDX4* bisulfite seq Rv1 (5′-AAACCTCTCCTCTATCTCC-3′). A nested PCR was performed on this PCR product using methylation insensitive primers *PRDX4* bisulfite seq Fw2 (5′- TAAATGTAGGTTTGGGATGG-3′) and *PRDX4* bisulfite seq Rv2 (5′- CCAACCCTACACAACTCCAA-3′). For sequencing, the PCR product was sub-cloned into TA cloning vector (Invitrogen, Breda, the Netherlands). DNA was isolated from individual colonies and sequenced using the M13Fw (5′-GTAAAACGACGGCCAG-3′) and the M13Rv (5′-CAGGAAACAGCTATGAC-3′) primers.

### Chromatin isolation

Ten million cells were suspended in 10 ml culture medium and cross-linked with 1% formaldehyde for 10 min at RT. The reaction was quenched with 130 mM glycine. Cells were washed twice with cold PBS and subsequently incubated on ice for 10 min with cold nuclei lysis buffer (50 mM Tris HCl, 10 mM EDTA, 1% SDS) to which 1 mM protease inhibitors (Sigmafast, Sigma Aldrich, St Louis, MO) and 4 mM PMSF (Sigma Aldrich, St Louis, MO) was freshly added. Lysed cells were subjected to 6 cycles of sonication for 20 sec to obtain fragments of 200–1000 bp (Sanyo Soniprep 150). After centrifugation (10 minutes, 10.000 g) at 4°C to remove cell debris, the chromatin-containing supernatant fraction was pre-cleared by incubation with 50 µg pre-immune serum (IgG from rabbit serum I8140, Sigma-Aldrich, St Louis, MO) for 30 min at 4°C followed by addition of 100 µl protein A magnetic beads (Invitrogen, Breda, the Netherlands) for 30 min at RT.

### Chromatin immunoprecipitation (ChIP)

For immunoprecipitation, protein A dynabeads (Invitrogen, Breda, the Netherlands) were pre-incubated with antibodies against H3K27me3 (07-449, Upstate Biotechnology Inc, Charlottesville, VA) or IgG pre-immune serum as control (Sigma Aldrich, St Louis, MO). Twenty-five µl beads and 2.5 µg antibody were incubated for 30 min at room temperature. Beads pre-coupled to respective antibodies were then added to the pre-cleared chromatin and incubated for 2 hrs at 4°C. Magnetic beads containing the chromatin complexes were collected with a magnet and sequentially washed (5×) with 500 µl: 1× low salt wash for 1 min (20 mM Tris-HCl, 150 mM NaCl, 0.1% SDS, 1% Triton X-100, 1 mM PMSF), 1× high salt wash for 1 min (20 mM Tris-HCl, 500 mM NaCl, 0.1% SDS, 1% Triton X-100, 2 mM EDTA, 1 mM PMSF), 1× LiCl wash for 5 min (10 mM Tris-HCl, 0.25 M LiCl, 1 mM EDTA, 1% IGEPAL, 1% deoxycholate, 1 mM PMSF) and 2× TE buffer for 1 min each (10 mM Tris HCl, 1 mM EDTA). ChIP samples were eluted with 200 µl elution buffer (25 mM Tris HCl, 10 mM EDTA, 0.5% SDS) at 65°C for one hr, subsequently 3.75 mM NaCl was added and incubated overnight at 65°C to reverse the cross-linking. After de-cross-linking, 2.4 µg proteinase K (Roche, Basel, Switzerland) was added for protein digestion. DNA was purified using a QIAquick PCR purification kit (Qiagen, Hilden, Germany).

Quantitative PCR of ChIP-enriched sequences was performed using SYBR Green PCR Master Mix (Applied Biosystems, Weiterstadt, Germany). The *MYT1* gene served as a positive control in H3K27me3 ChIP [26]. For *PRDX4*, a primer set for the predicted TSS was used: Fw (5′-CAAATGCAGGCTTGGGATGG-3′) and Rv (5′-CAGCGCCTCCATGACCACG-3′).
